# Latent profile analysis of adolescent quality of life and its associations with lifestyle behaviors

**DOI:** 10.3389/fpubh.2026.1910401

**Published:** 2026-10-14

**Authors:** Xiaomin Song, Zhiwen Yang

**Affiliations:** 1 Department of Pediatrics, Heping Hospital Affiliated to Changzhi Medical College, Changzhi, Shanxi, China; 2 School of Physical Education, Shandong University, Jinan, Shandong, China

**Keywords:** adolescent, latent profile analysis, lifestyle behaviors, multinomial logistic regression, quality of life

## Abstract

**Background:**

Adolescent quality of life (QoL) is critical for development, yet its heterogeneity is underexplored. We aimed to identify latent QoL profiles and examine associations with lifestyle behaviors.

**Methods:**

Latent profile analysis (LPA) was performed on 7,244 Chinese adolescents (49.3% boys) using the Quality of Life Scale for Children and Adolescents. Multinomial logistic regression tested links between QoL profiles and sleep duration, physical activity, screen time, ultra-processed foods (UPF), and minimally processed foods (MPF), adjusting for demographics.

**Results:**

Five profiles emerged: Low QoL (C1), Health-advantaged (C2), Moderate QoL (C3), Low Physical and Psychological Health (C4), and High QoL (C5). Relative to C3, longer sleep duration was associated with lower odds of C1 (OR = 0.83) and C2 (OR = 0.74), but higher odds of C4 (OR = 1.26) and C5 (OR = 1.27). Higher physical activity was associated with lower odds of C2 (OR = 0.71) and higher odds of C4 (OR = 1.41) and C5 (OR = 1.62). Screen time was associated with lower odds of C2 and higher odds of C4. Greater MPF intake was associated with lower odds of C1 (OR = 0.70) and C2 (OR = 0.61), and higher odds of C4 (OR = 1.78) and C5 (OR = 1.35). Higher UPF intake was associated with lower odds of C1 (OR = 0.25), C2 (OR = 0.54), and C5 (OR = 0.56), and higher odds of C4 (OR = 1.45).

**Conclusion:**

Relative to the Moderate QoL class, lifestyle behaviors showed distinct associations with QoL profiles. Sleep duration, physical activity, and MPF intake were associated with lower odds of the Health-advantaged class and higher odds of the Low Physical and Psychological Health and High QoL classes. UPF intake was inversely associated with Low QoL, Health-advantaged, and High QoL classes, and positively associated with the Low Physical and Psychological Health class. These findings highlight the heterogeneity of adolescent QoL and suggest that interventions should be tailored to specific QoL profiles.

## 1 Introduction

Quality of life (QoL) is defined as an individual’s perception of their current living situation. This perception is formed within the context of their culture and value system and is linked to an individual’s goals, expectations, standards, and concerns (1). As a key indicator of well-being, QoL reflects not only physical and mental health but also influences social adaptation and long-term development (2). Considerable evidence indicates that individuals with low QoL experience greater social and psychological stress (3, 4), manifested as poor sleep quality (5), unhealthy dietary patterns (6), and severe internalizing problems such as anxiety, obsessive-compulsive symptoms, and depression (7, 8). In contrast, individuals who maintain a high QoL exhibit better mental health, improved sleep quality (9, 10), and stronger social support (8), and superior academic performance (11).

Adolescence is a critical period for growth. During this life stage, individuals undergo rapid development in physical maturation, cognitive reshaping, and psychological growth (12). QoL not only affects adolescents’ current physical and mental health but may also have long-term effects on their adult development. Therefore, examining adolescent QoL and exploring its influencing factors holds both theoretical value and practical importance for promoting their overall development.

Among the various factors affecting the QoL, diet, sleep duration (SD), screen time (ST), and physical activity (PA) all play significant roles. Regarding diet, ultra-processed foods (UPF) and minimally processed foods (MPF) are closely related to adolescent physical and mental health (13). UPF often contain large amounts of sugar, colorings, additives, and saturated fats. Long-term intake not only increases obesity rates among adolescents (14, 15), but may also raise the risk of mental disorders (16), leading to lower QoL scores (17, 18). In contrast, MPF based on natural ingredients have a protective effect on adolescent QoL (18). Meanwhile, SD is essential for adolescent growth and development. Research consistently shows that insufficient SD or poor-quality sleep may reduce adolescent QoL (19), while adequate SD is a prerequisite for better QoL (20). Additionally, ST is one of the most notable behavioral patterns in adolescent lifestyles. Excessive ST not only displaces sleep and PA but also triggers social media stress and the risk of internet addiction, which harms mental health (21), and lowers QoL scores (20). Regular PA, as a core part of a healthy lifestyle, helps maintain healthy weight and cardiovascular function. It also promotes brain development through neural mechanisms (22), prevents mental disorders such as anxiety and depression (23), and is positively associated with high QoL (24).

However, although previous studies have examined the independent linear relationships between these lifestyle variables and adolescent QoL, most research has treated adolescents as a homogeneous group. This approach overlooks potential internal heterogeneity, meaning that different subgroups may show unique patterns and distribution characteristics across various QoL dimensions.

Latent Profile Analysis (LPA), as a person-centered method, can effectively identify latent heterogeneous subgroups within a population, thus providing a more precise depiction of the distribution of adolescent QoL. This study aims to use LPA to identify latent subgroups with distinct QoL patterns and further apply multinomial logistic regression to examine the associations between these subgroups and lifestyle factors. By identifying risk characteristics and protective factors for different QoL classes, this study seeks to offer new insights into the complex structure of adolescent QoL and to provide empirical evidence for developing more targeted intervention strategies.

## 2 Methods

### 2.1 Participants

Data for this study were obtained from the DYH (Database of Youth Health), a long-term longitudinal dataset focused on adolescent health. This dataset represents the publicly shared dataset on adolescent health and health-related behaviors in China. Data collection involved on-site testing and questionnaires, covering 186 middle schools in 17 cities of Shandong Province. Cross-sectional data from the 2020–2021 wave were used in this study.

This study was conducted in strict accordance with the ethical guidelines of the Declaration of Helsinki and was formally reviewed and approved by the Ethics Committee of Shandong University (Approval No. 20180517). Prior to survey administration, written informed consent was obtained from all participating adolescents as well as their parents or legal guardians. Participation was entirely voluntary, with students having the right to withdraw at any stage without consequence. Stringent protocols were implemented throughout data collection to ensure complete anonymity and confidentiality of all individual records. Standardized instructions were provided by trained field administrators to assist students during questionnaire completion.

A total of 8,179 completed questionnaires were initially retrieved. Participants with missing information on key study variables were excluded, resulting in a final analytical sample of 7,244 respondents (3,571 boys and 3,673 girls). To examine whether data attrition introduced selection bias, we compared included (n = 7,244) and excluded (n = 935) participants across demographic parameters (gender, residence, and parental education). No statistically significant or practically meaningful differences were identified between the two groups, confirming that sample attrition did not materially distort sample representativeness.

### 2.2 Measures

#### 2.2.1 Quality of life

In this study, the Quality of Life Scale for Children and Adolescents (QLSCA) was employed to assess the QoL of adolescents. This scale was specifically developed for Chinese children and adolescents and has been widely applied in previous research (25–27). The scale comprises 49 items hierarchically mapped onto 13 aspect-level facets across four overarching domains: (1) Psychosocial Function (PF): Peer relationships (PF1), Teacher-student relationships (PF2), Learning attitude (PF3), Academic stress (PF4), and Self-cognition (PF5). (2) Physical and Psychological Health (PPH): Physical health (PPH1), Mental health (PPH2), and Negative emotions (PPH3). (3) Living Environment (LE): Family environment (LE1), Peer environment (LE2), and School environment (LE3). (4) Satisfaction with Life (SWL): Life satisfaction (SWL1) and Freedom/autonomy (SWL2).

The total score of QoL was obtained by summing all item scores. Each item was rated on a four-point Likert scale, with higher scores indicating better QoL. The scale demonstrated satisfactory reliability and validity for the present sample, with Cronbach’s α = 0.922 and a Kaiser–Meyer–Olkin (KMO) measure of 0.965 (*p* < 0.001).

#### 2.2.2 Physical activity

PA was assessed using the Physical Activity Questionnaire for Children (PAQ-C), which has been widely used in China (28, 29). This questionnaire consists of ten items. The first item records the types and frequency of PA performed during leisure time. The second to seventh items evaluate the PA situation in various contexts, including physical education classes, recess, lunchtime, after school, evenings, and weekends. The eighth item records the overall PA performance in the past week, while the ninth item measures daily PA frequency during the same period. The tenth item is not included in the total score.

Each item was scored on a scale from 1 to 5, and the total score of the PAQ-C was calculated as the average of the first nine items, with higher scores indicating higher frequency of physical activity. The reliability and validity of the scale were also tested in the present sample, with Cronbach’s α = 0.879 and KMO = 0.966 (*p* < 0.001).

#### 2.2.3 Ultra-processed foods and minimally processed foods

Adolescents reported their intake of UPF and MPF over the past week by describing the types and frequency of consumption. Classification of UPF and MPF was based on the NOVA food classification system. UPF were categorized into six groups: processed meats, instant noodles, fast food, sweet or salty snacks and desserts, sugar-sweetened beverages, and fried foods. MPF were classified into 11 groups: leafy green vegetables; red or orange vegetables; potatoes; other vegetables; tubers; fruits; soy products; fresh meat; fish, shrimp, or other seafood; eggs; and milk (30).

The frequency of food intake was evaluated according to a five-level scoring system: the corresponding scores range from 1 to 5. Specifically, a score of 1 indicates no consumption per week; a score of 2 indicates 1–2 times per week; a score of 3 indicates 3–5 times per week (approximately every other day); a score of 4 indicates 6–7 times per week (once daily); and a score of 5 indicates 8 times or more per week (more than once a day).

#### 2.2.4 Screen time

Daily ST was evaluated by asking adolescents to report their average daily duration of non-academic electronic screen use (including smartphones, video games, computers, and television) over the preceding week. Responses were scored on a 7-point scale: 1 = none, 2 = <1 h/day, 3 = 1 h/day, 4 = 2 h/day, 5 = 3 h/day, 6 = 4 h/day, and 7 = ≥5 h/day (31).

#### 2.2.5 Sleep duration

All adolescents were asked to recall and record their average daily SD over the past week. Reports below 4 h or above 12 h per day were considered implausible and were excluded from the analysis. The retained SD values therefore ranged from 4 to 12 h per day.

#### 2.2.6 Covariates

Potential confounding variables were also collected, including gender, residential location, only-child status, and socioeconomic status (SES). Gender was coded as a binary variable (1 = boy; 2 = girl). Only-child status was classified into two categories (1 = only child; 2 = non-only children). Residential location was categorized as either urban or rural (1 = urban; 2 = rural). SES was measured using five items covering parental education level, parental occupation, and self-rated family economic status. The total score was calculated by summing the five items, with higher scores indicating higher SES (31), The observed total score ranged from 5 to 47, with Cronbach’s α = 0.714.

### 2.3 Sensitivity analyses

Several sensitivity analyses were conducted to assess the robustness of the latent profile solution, particularly the relatively small endpoint classes. First, the five-class LPA was re-estimated under a less restrictive residual-variance specification by allowing indicator variances to vary across latent classes. Second, multivariate outliers were identified using Mahalanobis distance based on the QoL indicators, and the LPA was repeated after excluding these observations. The resulting number and proportion of latent classes, classification quality, and overall profile patterns were compared with those of the primary five-class solution.

### 2.4 Statistical analysis

Descriptive statistics were computed using SPSS 27.0. Latent profile analysis was performed in Mplus 8.3 using the 49 individual QLSCA items as continuous profile indicators. Utilizing item-level indicators maximizes measurement precision by capturing subtle patterns of item endorsements that might be masked by composite scores. Following latent class extraction and enumeration, to facilitate substantive interpretation and align with the psychometric structure of the QLSCA, the 49 items were summarized into the 13 established facet scores by calculating the mean score of the constituent items for each facet. These 13 facet-level means were then used to substantively characterize the profiles and generate the profile plots.

LPA sequentially fits multiple latent class models, and the optimal model is determined by comparing multiple fitting indicators among the models. The main indices included: (1) the Akaike Information Criterion (AIC), Bayesian Information Criterion (BIC), and sample-size adjusted Bayesian Information Criterion (ABIC); (2) Entropy, which reflects the accuracy of classification; (3) the Lo–Mendell–Rubin likelihood ratio test (LMR) and the bootstrapped likelihood ratio test (BLRT). The statistical results of these two tests (*p* < 0.05) indicate that the k-class model has significantly better fit than the (k - 1)-class model. The final model was selected based on a combination of these statistical indicators and substantive interpretability (32, 33). All latent profile models were estimated using the robust maximum likelihood estimator (MLR) with 1,000 random starts and 250 final-stage optimizations (STARTS = 1,000 250; STITERATIONS = 50); convergence was verified by replicating the best log-likelihood across random start sets.

After selecting the optimal model, chi-square tests and analysis of variance (ANOVA) were used to examine differences in variables across groups. Multinomial logistic regression was then applied to examine associations between lifestyle behaviors and QoL class membership, with the Moderate QoL class as the reference category. Continuous predictors (SD, ST, MPF, UPF, PA) and SES were standardized to Z-scores. Gender, only-child status, and residence were entered as categorical covariates. Model fit was assessed using the likelihood ratio test and McFadden’s pseudo-R^2^. Multicollinearity was examined using variance inflation factors (VIFs). All VIFs were < 2.0.

## 3 Results

### 3.1 Demographic characteristics

As shown in Table 1, this study surveyed a total of 7,244 adolescents, including 3,571 boys (49.3%) and 3,673 girls (50.7%). Among them, 2,299 (31.74%) were only children, while 4,945 (68.26%) had siblings. The proportion of only-child boys (39.12%) was notably higher than that of only-child girls (24.56%). Regarding residential distribution, 2,829 participants (39.05%) resided in urban areas, and 4,415 (60.95%) lived in rural areas. In terms of paternal education, the largest group of adolescents reported that their fathers had completed junior high school (n = 2,895, 39.96%), followed by senior high school (n = 1,106, 15.27%). For maternal education, the most prevalent category was also junior high school (n = 2,705, 37.34%), with primary school being the second most common (n = 1,320, 18.22%).

**Table 1:** Characteristics of participants (*n* = 7,244).

Item	Characteristics	Gender (%)		All	χ^2^	*p*
		Boys	Girls			
Only child status	Non-only children	2,174(60.88)	2,771(75.44)	4,945(68.26)	177.252	0.000
	Only child	1,397(39.12)	902(24.56)	2,299(31.74)		
Resident	Urban	1,456(40.77)	1,373(37.38)	2,829(39.05)	8.753	0.003
	Rural	2,115(59.23)	2,300(62.62)	4,415(60.95)		
Father’s education	No education	86(2.41)	81(2.21)	167(2.31)	20.626	0.008
	Primary school	323(9.05)	355(9.67)	678(9.36)		
	Junior high school	1,374(38.48)	1,521(41.41)	2,895(39.96)		
	Technical school	343(9.61)	363(9.88)	706(9.75)		
	Vocational high school	168(4.70)	189(5.15)	357(4.93)		
	Senior high school	568(15.91)	538(14.65)	1,106(15.27)		
	Junior college	342(9.58)	311(8.47)	653(9.01)		
	Bachelor degree	288(8.06)	268(7.30)	556(7.68)		
	Postgraduate and above	79(2.21)	47(1.28)	126(1.74)		
Mother’s education	No education	173(4.84)	147(4.00)	320(4.42)	23.622	0.003
	Primary school	656(18.37)	664(18.08)	1,320(18.22)		
	Junior high school	1,288(36.07)	1,417(38.58)	2,705(37.34)		
	Technical school	313(8.77)	361(9.83)	674(9.30)		
	Vocational high school	123(3.44)	156(4.25)	279(3.85)		
	Senior high school	441(12.35)	424(11.54)	865(11.94)		
	Junior college	267(7.48)	264(7.19)	531(7.33)		
	Bachelor degree	241(6.75)	190(5.17)	431(5.95)		
	Postgraduate and above	69(1.93)	50(1.36)	119(1.64)		
Total		3,571	3,673	7,244		

Values are expressed as *n* (%). *p*-values were derived from Pearson’s chi-square tests.

### 3.2 Selection of the optimal latent profile model for quality of life

Table 2 reports the fit indices for the one- to six-class solutions. The AIC, BIC, and ABIC decreased monotonically as the number of latent classes increased, and the BLRT was significant for all models from the three-class to the six-class solution. Thus, these global fit indices favored more complex solutions and did not unambiguously identify a single optimal model. Model selection was therefore based on the LMR test, classification quality, substantive interpretability, and parsimony.

**Table 2:** Model fit indices for models with 1 to 6 classes.

Quality of life	LL	AIC	BIC	ABIC	Entropy	BLRT/P	LMR/P
1C	−431878.986	863953.972	864628.989	864317.567			
2C	−400589.553	801475.107	802494.520	802024.209	0.935		
3C	−385689.633	771775.267	773139.077	772509.877	0.964	*P* < 0.01	0.324
4C	−377346.609	755189.218	756897.424	756109.335	0.955	P < 0.01	0.093
5C	**−369370.687**	**739337.374**	**741389.977**	**740442.999**	**0.961**	***P* < 0.01**	***p* <0.01**
6C	−365737.748	732171.495	734568.495	733462.628	0.935	*P* < 0.01	0.762

For the selected five-class model, class sizes and proportions were: C1 = 143 (1.97%), C2 = 1,599 (22.07%), C3 = 3,663 (50.57%), C4 = 1,589 (21.94%), C5 = 250 (3.45%). LL: Log-likelihood; AIC: Akaike information criterion; BIC: Bayesian information criterion; ABIC: Adjusted Bayesian information criterion; BLRT: Bootstrapped likelihood ratio test; LMR: Lo–Mendell–Rubin Likelihood ratio test. Bolded content indicates specific fit indices of the selected LPA model.

The five-class solution was retained because it provided the best balance of these criteria. Specifically, the five-class solution had high entropy (0.961) and its LMR test was statistically significant (*p* < 0.01), whereas the LMR tests for the four-class model (*p* = 0.093) and the six-class model (*p* = 0.762) were not significant. Substantively, the five-class model yielded five theoretically coherent and clearly distinguishable QoL profiles. In contrast, the three-class model was considered too parsimonious because it collapsed distinct profiles, and the six-class solution was not preferred because its additional class did not add meaningful substantive separation beyond the five-class structure.

Taken together, the five-class solution was retained based on the combined evidence from statistical fit, classification quality, substantive interpretability, and parsimony. Its robustness was further evaluated in subsequent sensitivity analyses.

### 3.3 Definition of the optimal latent profile model for quality of life

As shown in Figure 1, based on the characteristics of the QoL of adolescents, a total of five potential classes were identified. These classes were, respectively, labeled as C1 to C5. The y-axis represents the mean scores of QoL of adolescents, while the x-axis corresponds to the 13 measurement items used in the LPA. The five latent classes were estimated using all 49 items of the QLSCA. For visual clarity and substantive interpretability.

**Figure 1:**
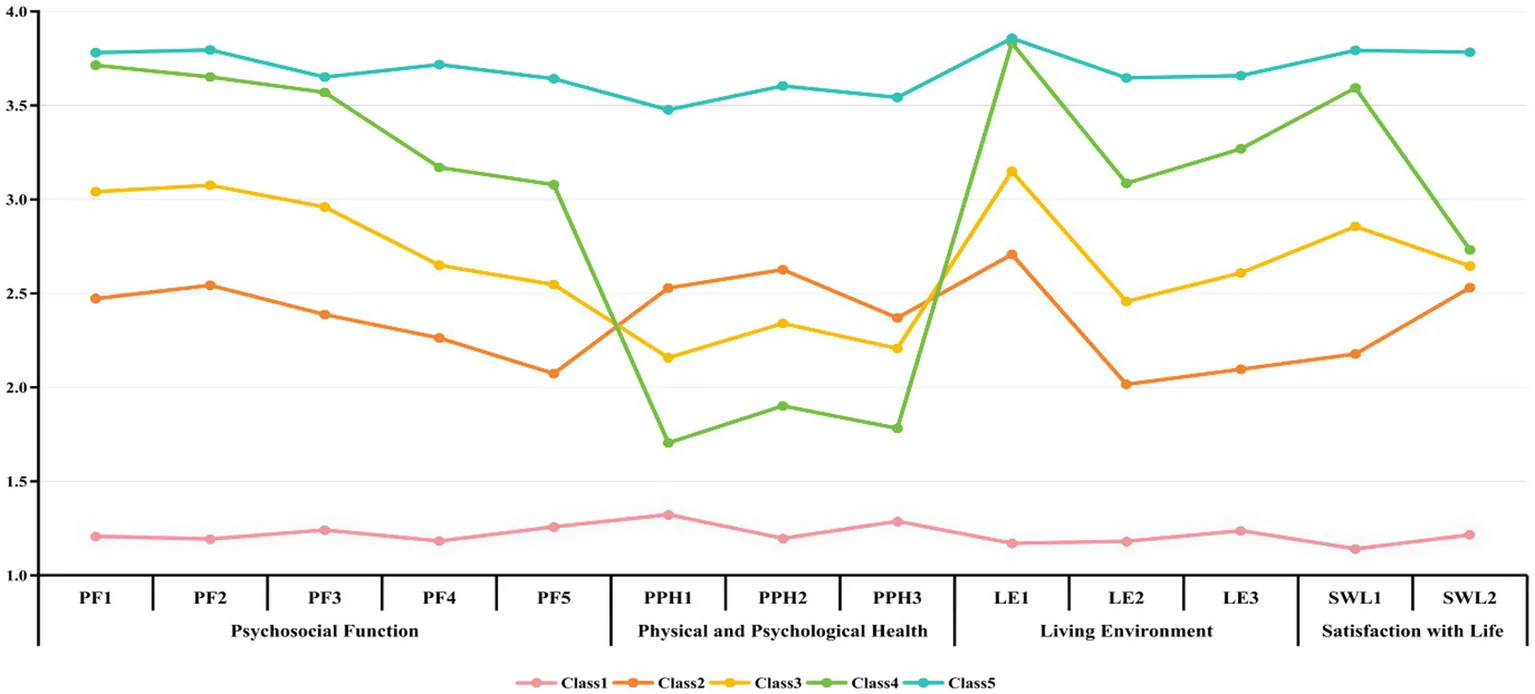
Analysis results of latent profile of adolescent quality of life. Class 1: Low QoL; Class 2: Health-advantaged; Class 3: Moderate QoL; Class 4: Low Physical and Psychological Health; Class 5: High QoL.

C1 was defined as the “Low QoL.” The adolescents in this group scored the lowest in all dimensions of QoL, representing the group with the poorest overall QoL.

C2 was defined as the “Health-advantaged.” The adolescents in this group scored second only to the “High QoL” on the physical and psychological health dimension, but their scores in other dimensions were very low, only higher than the “Low QoL” group. This pattern indicates that their physical and psychological health dimension performed exceptionally well compared to other dimensions.

C3 was labeled the “Moderate QoL.” The adolescents in this group scored at the medium level in all dimensions, ranking third among the five groups.

C4 was termed the “Low Physical and Psychological Health”. Adolescents in this class scored very low on the physical and psychological health dimension only second to the “Low QoL,” but their scores in the other three dimensions were very high, only lower than the groups with “High QoL”.

C5 was designated as the “High QoL”. The adolescents in this group achieved the highest scores in all dimensions, indicating that they are the group with the best QoL.

### 3.4 Differences in demographic characteristics across quality of life classes

As shown in Table 3, significant differences were observed across the five QoL classes with respect to gender (χ^2^ = 55.598, *p* < 0.001). In both the “Low QoL” and the “High QoL,” the proportion of boys reached 64.34 and 64.00% respectively, higher than the average of the overall sample of 49.30%. Conversely, the proportion of girls in these two groups was 35.66 and 36.00% respectively, lower than the overall average of 50.70%.

**Table 3:** Demographic differences across quality of life categories.

Item	Characteristics	Class of quality of life (%)					All	χ^2^/*F*	*p*
		C1	C2	C3	C4	C5			
Gender	Boys	92 (64.34)	727 (45.47)	1751 (47.80)	841 (52.93)	160 (64.00)	3,571 (49.30)	55.598	0.000
	Girls	51 (35.66)	872 (54.53)	1912 (52.20)	748 (47.07)	90 (36.00)	3,673 (50.70)		
Only child status	Non-only children	39 (27.27)	1,136 (71.04)	2,581 (70.46)	1,066 (67.09)	123 (49.20)	4,945 (68.26)	167.736	0.000
	Only child	104 (72.73)	463 (28.96)	1,082 (29.54)	523 (32.91)	127 (50.80)	2,299 (31.74)		
Resident	Urban	48 (33.57)	550 (34.40)	1,365 (37.26)	755 (47.51)	111 (44.40)	2,829 (39.05)	72.095	0.000
	Rural	95 (66.43)	1,049 (65.60)	2,298 (62.74)	834 (52.49)	139 (55.60)	4,415 (60.95)		
Socioeconomic status		27.45 ± 10.42	19.59 ± 7.53	20.07 ± 7.72	22.02 ± 8.32	23.42 ± 9.44	20.65 ± 8.06	57.591	0.000
Total		143	1,599	3,663	1,589	250	7,244		

Values are presented as n (%) or mean ± standard deviation. p-values were calculated using Pearson’s chi-square tests for categorical variables and one-way ANOVA for continuous SES. C1: Low QoL; C2: Health-advantaged; C3: Moderate QoL; C4: Low Physical and Psychological Health; C5: High QoL.

A significant association was also found between QoL class and only-child status (χ^2^ = 167.736, *p* < 0.001). In the “Low QoL,” the proportion of non-only children was only 27.27%, much lower than the overall average of 68.26%.

In terms of residence, there were also significant differences among different classes (χ^2^ = 72.095, *p* < 0.001). In the “Low Physical and Psychological Health”, the proportion of adolescents living in urban was 47.51%, significantly higher than the overall average of 39.05%.

### 3.5 Sensitivity analyses

Sensitivity analyses confirmed the robustness and structural stability of the five-class solution. After excluding 119 multivariate outliers, the analytical sample was reduced to 7,125 participants. The five-class structure was robustly retained with high classification precision (Entropy = 0.974). Based on most likely class membership, C1 to C5 comprised 96 (1.35%), 1,080 (15.16%), 4,673 (65.59%), 1,190 (16.70%), and 86 (1.21%) participants, respectively. Although the proportions of C1 and C5 decreased slightly compared to the baseline analysis, C1 continued to represent the lowest overall QoL profile and C5 the highest, with the overall five-profile configuration remaining qualitatively identical. Similarly, when relaxing the residual variance constraints across classes (*N* = 7,244), the five-class model converged cleanly (Entropy = 0.971). The profile ordering and substantive characteristics were fully preserved.

### 3.6 Differences in health behaviors across classes of quality of life

As presented in Table 4, there were significant differences in SD across the five classes of QoL (*F* = 76.21, *p* < 0.001). Adolescents in the High QoL class reported the longest SD (*M* = 7.68). Followed by the Low Physical and Psychological Health class (*M* = 7.50). In contrast, the Health-advantaged class had the shortest SD (*M* = 6.91).

**Table 4:** Differences of health behavior across quality of life categories.

Item	Class of quality of life					*F*	*p*
	C1 (*n* = 143)	C2 (*n* = 1,599)	C3 (*n* = 3,663)	C4 (*n* = 1,589)	C5 (*n* = 250)		
Sleep duration	7.10 ± 1.35	6.91 ± 1.05	7.23 ± 1.00	7.50 ± 1.04	7.68 ± 1.37	76.210	0.000
Screen time	5.78 ± 1.99	5.50 ± 1.78	5.99 ± 1.45	6.36 ± 1.18	5.58 ± 2.05	67.854	0.000
Minimally processed foods	42.18 ± 12.16	33.78 ± 8.30	36.01 ± 7.69	40.44 ± 8.09	43.27 ± 9.84	199.715	0.000
Ultra-processed foods	13.07 ± 6.53	20.74 ± 5.35	22.40 ± 4.53	22.92 ± 4.85	16.34 ± 6.86	241.095	0.000
Physical activity	2.07 ± 0.90	1.86 ± 0.62	2.04 ± 0.62	2.33 ± 0.70	2.67 ± 1.06	154.348	0.000

Values are presented as mean ± standard deviation. p-values were derived from one-way analysis of variance (ANOVA). C1: Low QoL; C2: Health-advantaged; C3: Moderate QoL; C4: Low Physical and Psychological Health; C5: High QoL.

There were also significant differences in ST among the groups (*F* = 67.85, *p* < 0.001). The Low Physical and Psychological Health class had the longest ST (*M* = 6.36), while the Health-advantaged class and the High QoL class reported the shortest ST (*M* = 5.50 and *M* = 5.58, respectively).

There were also significant differences in the levels of PA among the groups (*F* = 154.35, *p* < 0.001). The High QoL class had the highest level of PA (*M* = 2.67), followed by the Low Physical and Psychological Health class (*M* = 2.33), while the Health-advantaged class reported the lowest level (*M* = 1.86).

MPF also showed significant differences across classes (*F* = 199.72, *p* < 0.001). The High QoL class had the highest amount of MPF (*M* = 43.27), followed by the Low QoL class (*M* = 42.18), while the Health-advantaged class had the lowest intake (*M* = 33.78).

Significant differences were also observed in the consumption of UPF (*F* = 241.10, *p* < 0.001). The Low Physical and Psychological Health class reported the highest intake (*M* = 22.92), which was comparable to that of the Moderate QoL class (*M* = 22.40). In contrast, the Low QoLclass had the lowest intake (*M* = 13.07).

### 3.7 Associations between health behaviors and quality of life class membership

As shown in Figure 2, multinomial logistic regression was conducted with the Moderate QoL class as the reference category. This class represented the largest and mid-range QoL profile. Covariates included gender, only-child status, residential location, and SES; independent variables included SD, ST, MPF, UPF, and PA. The model was statistically significant, χ^2^(36) = 2,541.55, *p* < 0.001, with McFadden’s R^2^ = 0.146. Table 5 presents the ORs and 95% CIs for each QoL class relative to the Moderate QoL class. Since continuous predictors were standardized, ORs represent the change per 1-SD increase.

**Figure 2:**
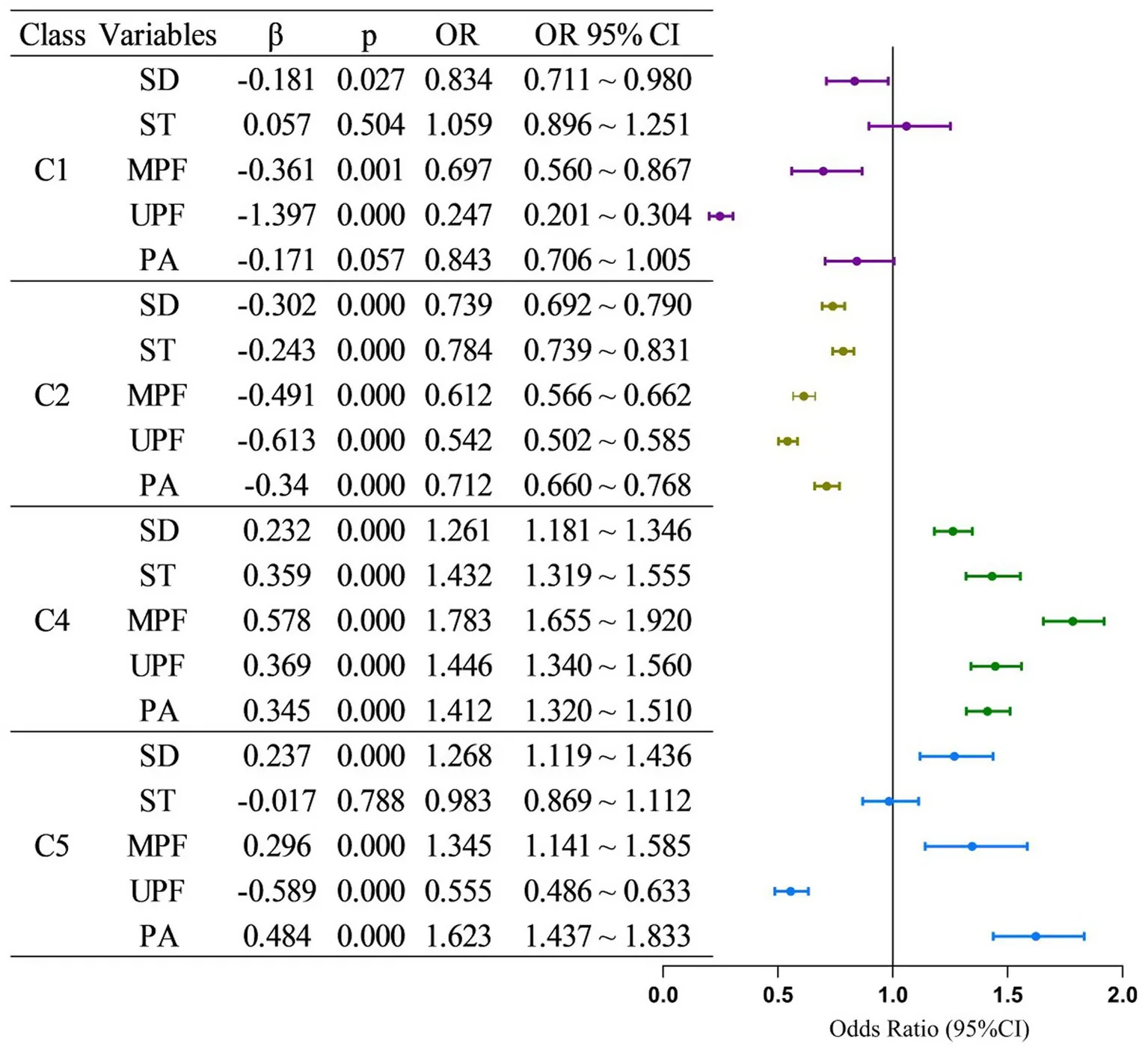
Test results of factors for different types of quality of life. Reference category = Moderate QoL (C3). OR, odds ratio; CI, confidence interval. C1: Low QoL; C2: Health-advantaged; C4: Low Physical and Psychological Health; C5: High QoL. Continuous predictors were standardized. SD: Screen time; ST: Sleep time; MPF: Minimally processed foods; UPF: Ultra-processed foods.

**Table 5:** Multinomial logistic regression predicting quality of life class membership.

Predictor	C1 OR (95% CI)	C2 OR (95% CI)	C4 OR (95% CI)	C5 OR (95% CI)
Gender	0.70 (0.48–1.03)	1.04 (0.92–1.19)	0.97 (0.85–1.11)	0.89 (0.67–1.18)
Only child status	3.33 (2.19–5.06)***	1.08 (0.93–1.26)	0.87 (0.75–1.01)	1.58 (1.19–2.12)**
Residence	2.18 (1.46–3.26)***	1.12 (0.97–1.29)	0.74 (0.64–0.85)***	0.91 (0.68–1.22)
SES	1.61 (1.35–1.91)***	0.97 (0.90–1.04)	1.13 (1.06–1.22)**	1.17 (1.02–1.35)*
Sleep duration	0.83 (0.71–0.98)*	0.74 (0.69–0.79)***	1.26 (1.18–1.35)***	1.27 (1.12–1.44)***
Screen time	1.06 (0.90–1.25)	0.78 (0.74–0.83)***	1.43 (1.32–1.56)***	0.98 (0.87–1.11)
Minimally processed foods	0.70 (0.56–0.87)**	0.61 (0.57–0.66)***	1.78 (1.66–1.92)***	1.35 (1.14–1.59)***
Ultra-processed foods	0.25 (0.20–0.30)***	0.54 (0.50–0.59)***	1.45 (1.34–1.56)***	0.56 (0.49–0.63)***
Physical activity	0.84 (0.71–1.01)	0.71 (0.66–0.77)***	1.41 (1.32–1.51)***	1.62 (1.44–1.83)***

Reference category = Moderate QoL (C3). OR = odds ratio; CI = confidence interval. C1: Low QoL; C2: Health-advantaged; C4: Low Physical and Psychological Health; C5: High QoL. Continuous predictors were standardized; McFadden’s R^2^ = 0.146. *p < 0.05; **p < 0.01; ***p < 0.001.

Relative to the Moderate QoL class, longer SD was associated with lower odds of being in the Low QoL class [OR = 0.83, 95% CI (0.71, 0.98), *p* = 0.027] and the Health-advantaged class [OR = 0.74, 95% CI (0.69, 0.79), *p* < 0.001], but higher odds of being in the Low Physical and Psychological Health class [OR = 1.26, 95% CI (1.18, 1.35), *p* < 0.001] and the High QoL class [OR = 1.27, 95% CI (1.12, 1.44), *p* < 0.001].

Longer ST was associated with lower odds of the Health-advantaged class [OR = 0.78, 95% CI (0.74, 0.83), *p* < 0.001] and higher odds of the Low Physical and Psychological Health class [OR = 1.43, 95% CI (1.32, 1.56), *p* < 0.001] relative to the Moderate QoL class. It was not significantly associated with the Low QoL class [OR = 1.06, 95% CI (0.90, 1.25), *p* = 0.504] or the High QoL class [OR = 0.98, 95% CI (0.87, 1.11), *p* = 0.788].

Higher MPF intake was associated with lower odds of the Low QoL class [OR = 0.70, 95% CI (0.56, 0.87), *p* = 0.001] and the Health-advantaged class [OR = 0.61, 95% CI (0.57, 0.66), *p* < 0.001], but higher odds of the Low Physical and Psychological Health class [OR = 1.78, 95% CI (1.66, 1.92), *p* < 0.001] and the High QoL class [OR = 1.35, 95% CI (1.14, 1.59), *p* < 0.001].

Higher UPF intake was associated with lower odds of the Low QoL class [OR = 0.25, 95% CI (0.20, 0.30), *p* < 0.001], the Health-advantaged class [OR = 0.54, 95% CI (0.50, 0.59), *p* < 0.001], and the High QoL class [OR = 0.56, 95% CI (0.49, 0.63), *p* < 0.001], but higher odds of the Low Physical and Psychological Health class [OR = 1.45, 95% CI (1.34, 1.56), *p* < 0.001].

Higher PA was associated with lower odds of the Health-advantaged class [OR = 0.71, 95% CI (0.66, 0.77), *p* < 0.001] and higher odds of the Low Physical and Psychological Health class [OR = 1.41, 95% CI (1.32, 1.51), *p* < 0.001] and the High QoL class [OR = 1.62, 95% CI (1.44, 1.83), *p* < 0.001] relative to the Moderate QoL class. PA was not significantly associated with the Low QoL class [OR = 0.84, 95% CI (0.71, 1.01), *p* = 0.057].

## 4 Discussion

This study used LPA to identify five distinct latent classes of QoL: the “Low QoL” group (C1), the “Health-advantaged” group (C2), the “Moderate QoL” group (C3), the “Low Physical and Psychological Health” group (C4), and the “High QoL” group (C5). Multinomial logistic regression, with C3 as the reference category, was used to examine the associations between lifestyle behaviors and QoL class membership. The results revealed a nuanced pattern: SD, PA, and MPF intake were generally associated with lower odds of the Health-advantaged class (C2) and higher odds of the Low Physical and Psychological Health (C4) and High QoL (C5) classes. UPF intake showed a distinct pattern, being inversely associated with C1, C2, and C5, but positively associated with C4. SD and MPF intake were also associated with lower odds of C1 relative to C3. These findings underscore the heterogeneity of adolescent QoL and suggest that lifestyle behaviors may relate differently to specific QoL profiles.

PA was associated with lower odds of membership in the C2 and higher odds of membership in the C4 and C5 classes, relative to the C3. This is consistent with previous research showing that regular PA has been associated with better physical function and mental health (34, 35), and is positively associated with high QoL (36). Furthermore, PA has been linked to better brain function and memory (37, 38), and to better self-learning ability in adolescents (39).

Longer SD was associated with lower odds of C1 and C2, but higher odds of C4 and C5, relative to C3. The positive association with C5 aligns with evidence that adequate sleep has been associated with better QoL. The negative associations with C1 and C2 suggest that shorter sleep is more characteristic of the Low QoL and Health-advantaged classes. The positive association with C4 may reflect that longer sleep is associated with underlying health problems or a distinct sleep pattern in this class. Previous studies have reported that both short and long sleep can be associated with lower QoL (40–42), but our findings suggest that the relationship depends on the QoL profile.

ST was associated with lower odds of C2 and higher odds of C4 relative to C3. Excessive ST may displace sleep and PA and has been linked to poorer mental health (21). The positive association with C4 may reflect that adolescents with low physical and psychological health spend more time on screens, possibly as a coping mechanism. The lack of association with C5 and C1 suggests that the relationship between ST and QoL is not uniform across profiles.

UPF intake exhibited a distinctive pattern. Relative to C3, higher UPF intake was associated with lower odds of C1, C2, and C5, but higher odds of C4. This diverges from conventional linear assumptions that UPF is uniformly detrimental. From a physical perspective, long-term UPF intake has been associated with increased the risk of diabetes, hypertension, and obesity (43, 44), and with inflammation, which in turn has been linked to various chronic diseases (45, 46). From a psychological perspective, UPF has been associated with depression and anxiety in adolescents and may also lead to sleep disturbances and suicidal ideation (47, 48), all of which are closely linked to adolescent QoL. However, the inverse association with C1 and C5 suggests that UPF intake is not simply a marker of poor QoL. The positive association with C4 is particularly interesting. C4 had the highest UPF intake, followed by C3, while C1 had the lowest. The positive association between UPF and C4 is not easily explained by SES. C4 had higher SES than C2 and C3, and C4 also had the highest UPF intake. It is also possible that the low cost and high energy density of UPF contribute to this association (49).

MPF intake was associated with lower odds of C1 and C2, and higher odds of C4 and C5, relative to C3. The inverse association with C1 is notable: although C1 had relatively high raw MPF intake, the adjusted model showed that higher MPF was associated with lower odds of C1. This suggests that the raw difference may be confounded by other factors, such as SES. For instance, excessive parental control over children’s diets for health reasons, such as pressuring or restricting eating, may deprive adolescents of dietary autonomy (50, 51) and contribute to psychological problems. Additionally, material advantages provided by parents and family stress can lead to serious psychological issues in adolescents, such as depression, anxiety, and substance abuse (52, 53). This finding highlights the need to consider family context when interpreting dietary disparities in adolescent QoL. Future research should further examine this relationship using longitudinal designs.

### 3.1 Practical and clinical implications

The five QoL profiles suggest that adolescent well-being is heterogeneous and that uniform health promotion is unlikely to fit all students. In practice, schools and primary care providers could use profile-specific screening. Adolescents in the Low QoL class should be prioritized for broad assessment and support addressing mental health, sleep, diet, physical activity, and family or school difficulties. Those in the Low Physical and Psychological Health class may need clinical review for physical or mental health problems alongside lifestyle counseling, even though their other QoL domains are relatively high. The Health-advantaged class may appear physically and mentally well but report poorer functioning in other QoL domains, so psychosocial and environmental issues should not be overlooked. The lifestyle findings point to sleep duration, physical activity, and diet as potential intervention targets, but the direction of associations varies by profile, reinforcing the need for tailored approaches.

### 3.2 Limitations

Several limitations remain and should be addressed in future research. First, this study used cross-sectional survey data, so causal or temporal relationships between lifestyle variables and latent QoL classes cannot be inferred. Future research should employ longitudinal designs to further examine dynamic predictive effects. Second, all measures in this study were based on adolescent self-report questionnaires. Future studies should incorporate more objective measurement data. Third, the analysis used most likely class membership rather than a formal three-step approach; however, entropy and average posterior probabilities indicated high classification accuracy. Finally, data were collected during the COVID-19 pandemic, which may have influenced lifestyle behaviors and QoL.

## 4 Conclusion

This study identified five heterogeneous subgroups of adolescent QoL using LPA. Relative to the Moderate QoL class, longer SD, higher PA, and greater MPF intake were associated with lower odds of the Health-advantaged class and higher odds of the Low Physical and Psychological Health and High QoL classes. UPF intake was inversely associated with the Low QoL, Health-advantaged, and High QoL classes, but positively associated with the Low Physical and Psychological Health class. These findings highlight the heterogeneity of adolescent QoL and suggest that interventions should be tailored to specific profiles.

## Data Availability

Publicly available datasets were analyzed in this study. This data can be found at: https://www.ncmi.cn/phda/dataDetails.do?id=CSTR:17970.11.A0031.202107.209.V1.0.
